# Global burden of disease from high-sodium diets, 1990–2021: analysis of GBD 2021 data

**DOI:** 10.3389/fnut.2025.1617644

**Published:** 2025-07-02

**Authors:** Yanwu Nie, Naibo Wang, Mingzhu Huang, Yuanzhi Li, Yuanan Lu, Hui Li, Lei Wu

**Affiliations:** ^1^School of Public Health, Jiangxi Provincial Key Laboratory of Disease Prevention and Public Health, Jiangxi Medical College, Nanchang University, Nanchang, China; ^2^Jiangxi Provincial Center for Patriotic Health and Health Promotion, Nanchang, China; ^3^Office of Public Health Studies, University of Hawaii at Manoa, Honolulu, HI, United States; ^4^Jiangxi Provincial Health Commission Key Laboratory of Pathogenic Diagnosis and Genomics of Emerging Infectious Diseases, Nanchang Center for Disease Control and Prevention, Nanchang, China

**Keywords:** GBD2021, high sodium intake, global trend, cardiovascular diseases, global burden

## Abstract

**Objectives:**

This study aimed to assess the global, regional, and national burden of disease attributable to high-sodium diets from 1990 to 2021.

**Methods:**

Data on the global high-sodium diet-related disease burden were obtained from the Global Burden of Disease 2021 (GBD2021). A Bayesian age-period-cohort model (BAPC) was used to project trends from 2022 to 2040.

**Results:**

Between 1990 and 2021, both the age-standardized death rate (ASDR) and age-standardized disability-adjusted life years rate (ASYR) attributable to high-sodium diets declined. The burden varied across Social Development Index (SDI) levels, regions, and countries, with Central Europe, East Asia, and Southeast Asia experiencing the highest burden. At the national level across 204 countries, the Republic of Bulgaria recorded the highest ASDR and ASYR, while the Islamic Republic of Pakistan experienced the most rapid increases in both ASDR and ASYR. Additionally, the ASDR and ASYR increased with age and were consistently higher in men than in women. The GBD2021 reported 8 diseases attributed to high-sodium diets, arranged in descending order of disease burden as follows: stroke, Ischemic heart disease, Hypertensive heart disease, Stomach cancer, Chronic kidney disease, Atrial fibrillation and flutter, Aortic aneurysm, Lower extremity peripheral arterial disease. The BAPC prediction results showed that the ASDR and ASYR of high-sodium diets would decrease by 23.28 and 19.46%, respectively, from 2022 to 2040.

**Conclusions:**

The global disease burden due to high-sodium diets has decreased over the past three decades, though disparities remain. Targeted investigations are urgently needed, particularly in high-burden regions, to further reduce the health impacts of excessive sodium intake.

## 1 Introduction

Sodium is an essential nutrient that plays a vital role in numerous physiological processes, including the regulation of water and electrolyte balance, acid-base homeostasis, and proper functioning of the heart and nervous system (1). A 2023 study by the World Health Organization (WHO) reported that the global average daily sodium intake was 4,310 mg/day—more than double the WHO recommended limit of 2,000 mg/day. GBD 2021 estimates indicate that excessive sodium intake is associated with ~1.86 million deaths annually. High dietary sodium consumption significantly increases the risk of various chronic non-communicable diseases. Research by Wang et al. (2) found that individuals with high sodium intake have a higher risk of cardiovascular disease (CVD); specifically, each 6-gram increase in sodium intake is associated with a 1% increase in CVD risk. An 8-year follow-up study also demonstrated that, after adjusting for confounding factors, higher sodium intake was linked to an increased risk of cardiovascular disease (3). Furthermore, a meta-analysis involving 261,732 participants reported a positive correlation between sodium intake and stroke risk (4). Wu et al. (5) also found a significant association between high salt consumption and an increased risk of gastric cancer. Another meta-analysis of randomized clinical trials showed that reducing sodium intake, increasing potassium intake, and using potassium-enriched salt substitutes in the diet can effectively lower blood pressure (6). Research by Yang et al. (7) further supports that lower sodium intake may help prevent major cardiovascular events. Additionally, findings from the 2019 Global Burden of Disease (GBD) study indicated a link between high-sodium diets and chronic kidney disease, with an estimated 45,530 global deaths annually attributed to high sodium-induced kidney disease (8). Overall, substantial evidence suggests that high dietary sodium intake is associated with multiple adverse health outcomes. Further analysis is warranted to understand the distribution of disease burden attributable to high sodium consumption, which could inform and guide the development of targeted public health policies.

According to the GBD 2019 study, dietary risks ranked third among all secondary risk factors contributing to GBD (9). Another research on dietary risks indicated that in 2017, Over half of diet-related deaths were attributed to excessive sodium intake (10). To date, only one GBD study has specifically examined the impact of high sodium intake from 1990 to 2019 (11), with a primary focus on regional variations and the effects of population growth and aging on sodium-related mortality. However, that study did not examine differences across gender and age groups or explore specific disease types associated with high sodium intake. Therefore, despite these insights, there remains a pressing need for updated and comprehensive data to inform current health policies and intervention strategies.

To address this gap, the present research utilized data from the GBD 2021, covering the period from 1990 to 2021. This study analyzed the disease burden attributable to high-sodium diets across different SDI levels, regions, countries, genders, and age groups. Furthermore, this paper projects future trends in age-standardized death rate and age-standardized disability-adjusted life years rate due to high-sodium diets through 2040.

## 2 Materials and methods

### 2.1 Data resources and collection

Data on high-sodium diets were obtained from the Global Burden of Disease Study Database in 2021 (https://vizhub.healthdata.org/gbd-results/), which provides global, regional, and national data on 88 risk factors from 1990 to 2021 (12). This study specifically used global data on high-sodium diets from 1990 to 2021. The variables collected include gender, age, region, country, Social Development Index, number of deaths, number of disability-adjusted life years (DALYs), age-standardized death rate (ASDR), and age-standardized disability-adjusted life years rate (ASYR).

### 2.2 Social development index (SDI)

The SDI is a composite measure that reflects a country's or region's level of development based on fertility rates, education levels, and per capita income. The SDI values range from 0 to 1, with higher values indicating higher levels of social and economic development (13). To better examine the relationship between high-sodium diets and development, this study categorized countries and regions into five SDI groups: low SDI, low-middle SDI, middle SDI, middle-high SDI, and high SDI.

### 2.3 Calculation of estimated annual percentage change (EAPC)

The EAPC is a commonly used metric for evaluating trends in age-standardized rates over a specific period (14). In this study, EAPC and its 95% confidence interval were calculated to assess the temporal trends of ASDR and ASYR attributable to high-sodium diets from 1990 to 2021. The formula used for EAPC calculation is as follows (15):


$$\begin{array}{l} \text{Ln(y)}=\alpha \text{+}\beta \text{(x)+}\varepsilon \text{,}\\ \text{EAPC}=100\%\;\times \left(\text{exp}\left(\beta \right)-1\right) \end{array}$$

Where “y” represents a rate (such as incidence rate, mortality rate, etc.), “x” represents the year, “β” is the slope of the trend segment, “α” is the intercept, and “ε” is the error term. If both the EAPC and its 95% CI value are >0, it can be considered that the corresponding age-standardized rate shows an upward trend; conversely, if both the EAPC and its 95% CI value are < 0, it is considered that the corresponding age-standardized rate shows a downward trend.

### 2.4 Bayesian age-period-cohort (BAPC) model

This study employed the BAPC model to predict the global disease burden attributable to high-sodium diets from 2022 to 2040 (16, 17). The BAPC model is an age-period-cohort model within the Bayesian framework. It treats all unknown parameters as random variables, assigning appropriate prior distributions, and does not rely on explicit parameter settings. The model estimates the posterior distribution by incorporating prior information about the unknown parameters based on the posterior distribution. The BAPC model can be expressed as:


$$\begin{array}{l} \text{ln}\left(\lambda_{ij}\right)=\mu +\alpha_{i}+\beta_{j}+\gamma_{k}+Z_{ij}, \end{array}$$

Among them, μ is the intercept, represents the age effect, β_*j*_ represents the period effect, γ_*k*_ represents the cohort effect, and Z_ij_ represents the unstructured variation parameter, adopting a Gaussian normal distribution with a mean of 0.


$$\begin{array}{l} {\text{Z}}_{ij}\sim \;N\left(0,k_{z}^{-1}\right) \end{array}$$

The BAPC model is implemented within the integrated nested Laplace approximation (INLA) framework for Bayesian inference. Compared to the traditional Markov chain Monte Carlo (MCMC) model, the INLA-based method has lower computational complexity and avoids common issues such as poor mixing and convergence in MCMC, while offering improved accuracy (16, 17).

### 2.5 Statistical analysis

In this study, the EAPC was used to evaluate trends across different SDI levels, regions, countries, and genders from 1990 to 2021. The BAPC model was used to predict the disease burden attributable to high-sodium diets from 2022 to 2040. All statistical analyses and data visualization were conducted using R version 4.3.1.

## 3 Results

### 3.1 Global level

**Figure 1:**
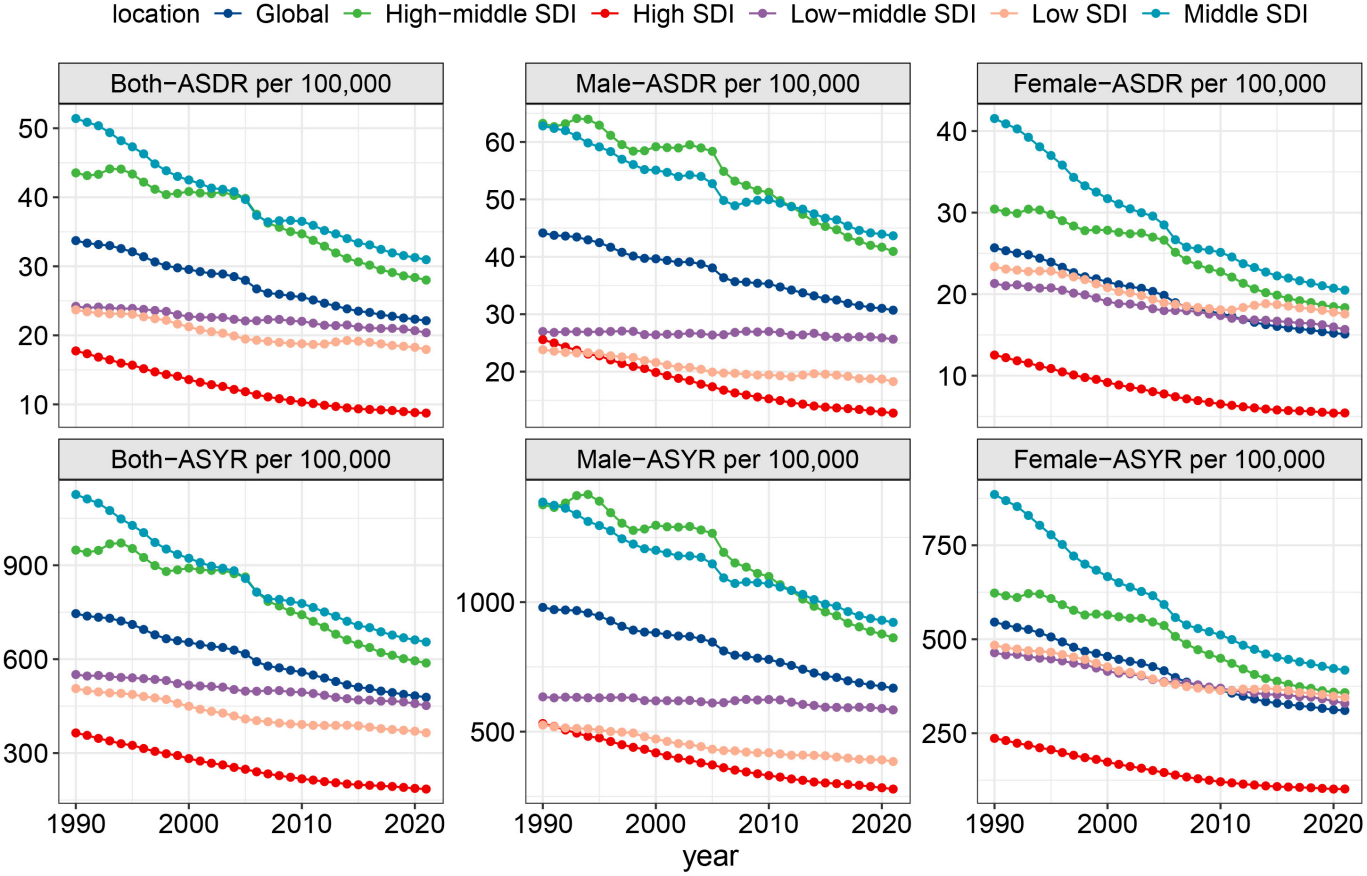
ASDR and ASYR of high-sodium diets at global and different SDI levels, 1990–2021.

In 2021, the number of deaths attributed to high-sodium diets was 1,857,695.59 (95% UI: 367,760.85 to 4,251,577.09), representing a 52.14% increase compared to 1990. Despite the substantial rise in absolute deaths, the ASDR per 100,000 population showed a decreasing trend, dropping from 33.72 (7.99 to 76.75) in 1990 to 22.12 (4.25 to 50.87) in 2021. The EAPC for ASDR was −1.44 (−1.48 to −1.4). In the same year, the number of DALYs attributable to high-sodium diets reached 41,275,914.09 (9,297,839.45 to 91,456,678.48), making a 40.50% increase from 1990. Similar to ASDR, ASYR also declined, falling from 745.54 per 100,000 population (199.38 to 1624.22) in 1990 to 478.29 (105.88 to 1064.85) in 2021, with an EAPC of −1.53 (−1.57 to −1.48) (Figure 1, Table 1, Supplementary Table S1).

**Table 1:** ASDR and ASYR trends in global, SDI and regional high–sodium diets, 1990–2021.

**Location**	**EAPC of ASDR (95%CI)**	**EAPC of ASYR (95%CI)**
Global	−1.44 (−1.48 to −1.40)	−1.53 (−1.57 to −1.48)
**SDI**		
High SDI	−2.39 (−2.49 to −2.29)	−2.28 (−2.37 to −2.18)
High–middle SDI	−1.58 (−1.72 to −1.44)	−1.74 (−1.89 to −1.59)
Middle SDI	−1.67 (−1.73 to −1.61)	−1.79 (−1.84 to −1.73)
Low–middle SDI	−0.54 (−0.56 to −0.51)	−0.63 (−0.66 to −0.61)
Low SDI	−0.91 (−1.02 to −0.80)	−1.11 (−1.2 to −1.02)
**Regional**		
Andean Latin America	−1.76 (−1.88 to −1.63)	−1.8 (−1.93 to −1.66)
Australasia	−3.45 (−3.57 to −3.34)	−3.43 (−3.59 to −3.27)
Caribbean	−1.45 (−1.60 to −1.29)	−1.14 (−1.31 to −0.98)
Central Asia	−1.94 (−2.14 to −1.74)	−2.35 (−2.58 to −2.11)
Central Europe	−2.38 (−2.46 to −2.30)	−2.61 (−2.7 to −2.51)
Central Latin America	−1.38 (−1.52 to −1.25)	−1.34 (−1.48 to −1.21)
Central Sub–Saharan Africa	−0.13 (−0.19 to −0.07)	−0.27 (−0.33 to −0.22)
East Asia	−1.76 (−1.85 to −1.68)	−1.88 (−1.95 to −1.81)
Eastern Europe	−1.64 (−2.12 to −1.16)	−1.68 (−2.21 to −1.15)
Eastern Sub–Saharan Africa	−1.87 (−1.98 to −1.77)	−2.17 (−2.29 to −2.05)
High–income Asia Pacific	−4.51 (−4.73 to −4.30)	−4.46 (−4.66 to −4.26)
High–income North America	−0.38 (−0.48 to −0.27)	0.21 (0.10 to 0.33)
North Africa and Middle East	−1.37 (−1.44 to −1.30)	−1.48 (−1.53 to −1.42)
Oceania	−0.86 (−0.92 to −0.80)	−0.72 (−0.79 to −0.64)
South Asia	0.17 (0.07 to 0.26)	0.14 (0.06 to 0.23)
Southeast Asia	−1.35 (−1.41 to −1.29)	−1.53 (−1.60 to −1.46)
Southern Latin America	−2.14 (−2.26 to −2.03)	−2.25 (−2.35 to −2.14)
Southern Sub–Saharan Africa	−0.04 (−0.4 to 0.32)	−0.4 (−0.75 to −0.06)
Tropical Latin America	−2.6 (−2.65 to −2.54)	−2.62 (−2.69 to −2.56)
Western Europe	−2.67 (−2.8 to −2.55)	−2.75 (−2.89 to −2.62)
Western Sub–Saharan Africa	−0.25 (−0.33 to −0.17)	−0.32 (−0.40 to −0.23)

### 3.2 Regional level

The global burden due to high-sodium diets exhibits marked regional disparities, closely related to SDI levels. In 2021, the Middle SDI region reported the highest ASDR and ASYR, at 30.96 per 100,000 population (6.83 to 67.93) and 654.82 per 100,000 population (168.32 to 1371.87) respectively. In contrast, the high SDI region recorded the lowest ASDR and ASYR, at 8.74 per 100,000 population (1.02 to 23.20) and 185.04 per 100,000 population (24.12 to 468.47) (Figure 1 and Table 1). Across all SDI regions, both ASDR and ASYR demonstrated a declining trend. The high SDI region experienced the most rapid decline, with EAPCs of −2.39 (−2.49 to −2.29) for ASDR and −2.28 (−2.37 to −2.18) for ASYR (Table 1, Supplementary Tables S1, S2).

In 2021, at the regional level, Central Europe reported the highest ASDR at 44.61 per 100,000 population (95% UI: 12.63 to 84.03), followed by East Asia at 42.96 (95% UI: 11.32 to 87.40), and Southeast Asia at 38.84 (95% UI: 6.83 to 81.73). In terms of ASYR, East Asia has the highest-burden at 890.63 per 100,000 population (95%UI: 294.06 to 1,700.52), followed by Southeast Asia at 856.53 (95%UI: 166.98 to 1,769.38), and Central Europe at 797.07 (95%UI: 231.80 to 1,500.36). While most regions showed a declining trend in both ASDR and ASYR from 1990 to 2021, an exception was observed in South Asia, where the EAPC of ASDR exhibited a slight upward trend at 0.17 (95% CI: 0.07 to 0.26). Additionally, the ASYR exhibited an increasing trend in both High-income North America and South Asia, with EAPCs of 0.21 (0.1 to 0.33) and 0.14 (0.06 to 0.23), respectively (Table 1, Supplementary Tables S1, S2).

### 3.3 National level

**Figure 2:**
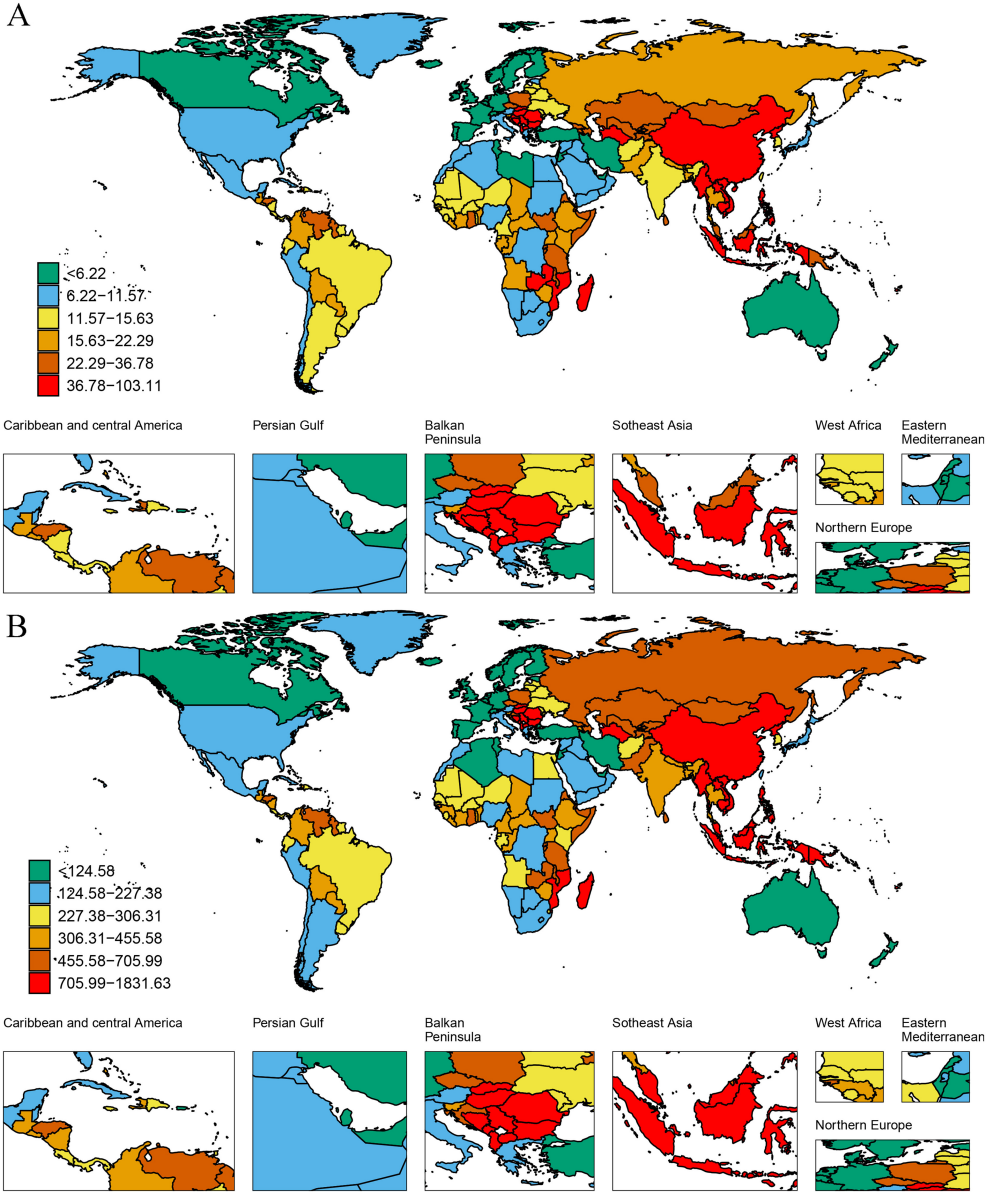
The distribution of ASDR **(A)** and ASYR **(B)** caused by high-sodium diets in 204 countries and territories in 2021.

In 2021, at the national level, among 204 countries analyzed, the Republic of Bulgaria recorded the highest ASDR at 103.11 per 100,000 population (32.89 to 182.22), followed by North Macedonia at 91.88 (26.02 to 169.06), and Montenegro at 76.37 (21.70 to 139.98). Similarly, the Republic of Bulgaria had the highest ASYR at 1,831.62 per 100,000 population (588.88 to 3,263.68), followed by the Republic of Nauru at 1,450.34 (142.30 to 3634.50), and North Macedonia at 1,433.91 (401.31 to 2,659.94). Furthermore, 29 countries exhibited an increasing trend in ASDR, while 24 countries showed an upward trend in ASYR. Notably, the Islamic Republic of Pakistan experienced the most rapid increases in both, with EAPCs of 1.76 (1.52 to 1.99) for ASDR and 1.78 (1.54 to 2.02) for ASYR (Figure 2, Supplementary Table S3).

### 3.4 Age and sex patterns

**Figure 3:**
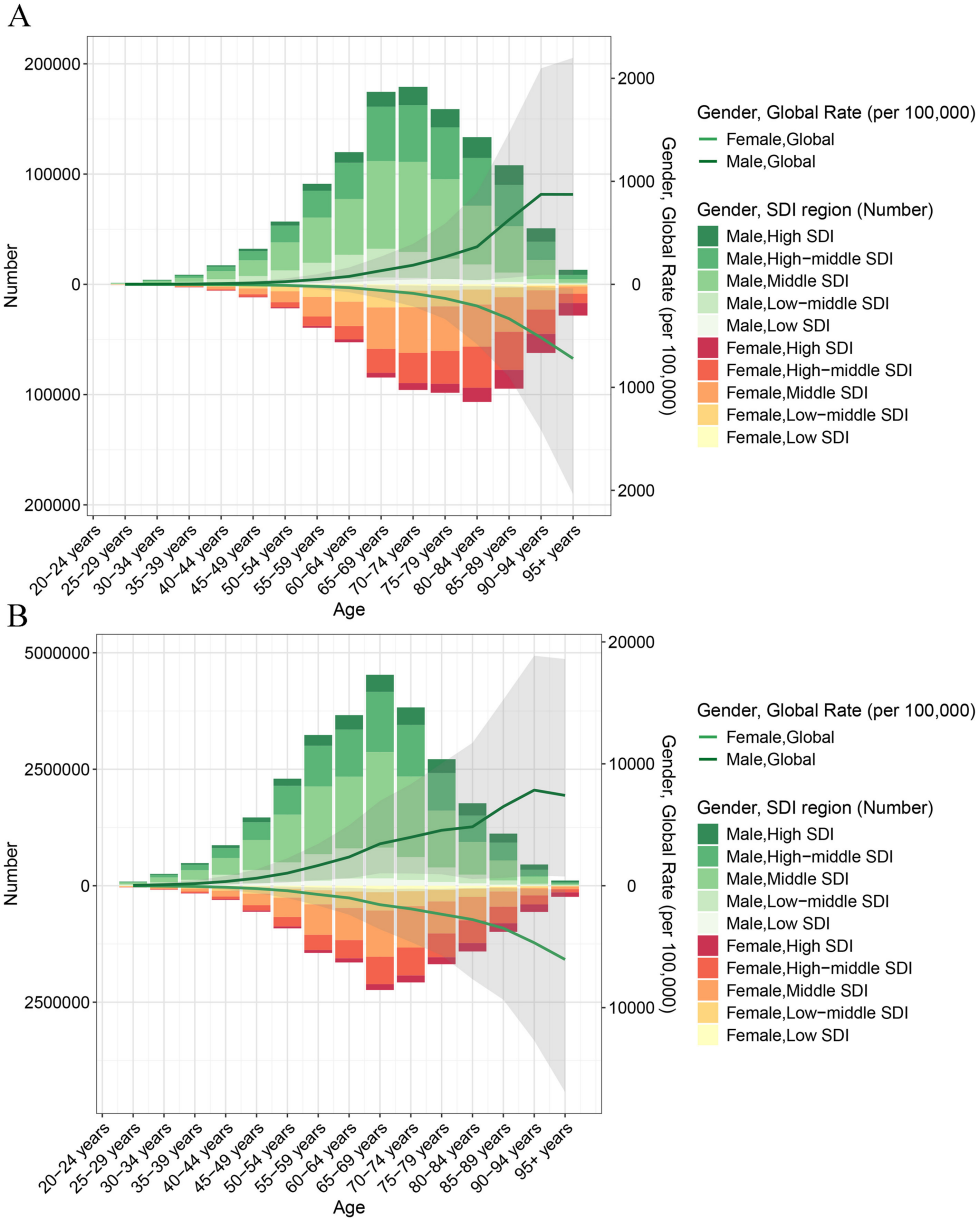
Age-specific ASDR **(A)** and ASYR **(B)** of high-sodium diets in different SDI regions in 2021.

**Figure 4:**
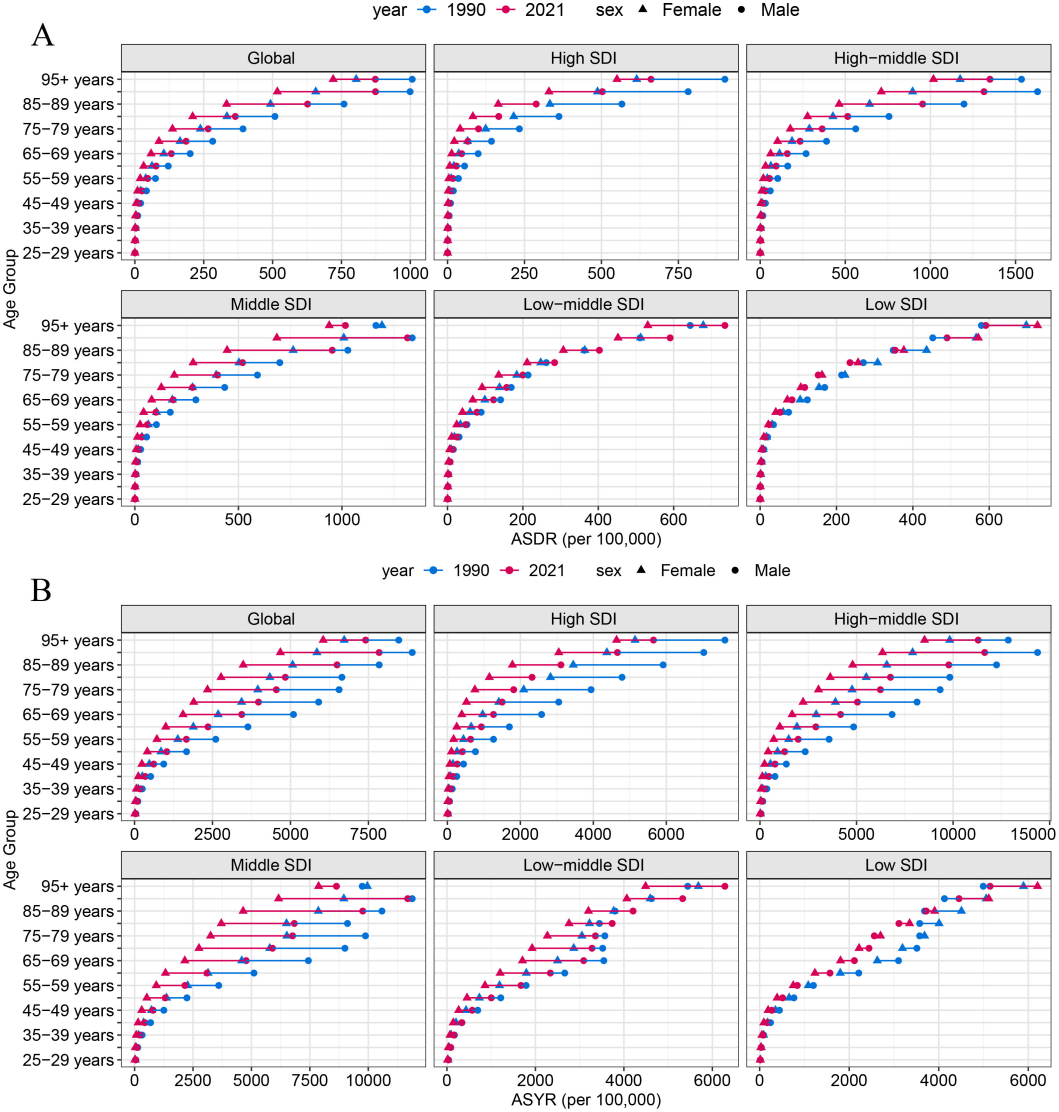
ASDR **(A)** and ASYR **(B)** of high-sodium diets by sex, age group, and social development index, 1990 and 2021.

In 2021, both the ASDR and ASYR attributable to high-sodium diets showed a gradual increase with age (Figure 3). Prior to the 40–44 age group, no significant differences were observed between males and females. However, beginning at the age of 45, both ASDR and ASYR were higher in males than in females, with the gender gap widening progressively with age and peaking in the 90–94 age group (Figure 4). Interestingly, in low SDI regions, the ASDR and ASYR were higher in females than in males, in contrast to the other four SDI regions, which followed the global pattern of higher burden in males.

### 3.5 The burden of different diseases caused by high-sodium diets

Table 2 shows the ASDR and ASYR for eight diseases due to high-sodium diets in 2021. Ranked from highest to lowest, the diseases with the greatest burden are as follows: Stroke, Ischemic heart disease, Hypertensive heart disease, Stomach cancer, Chronic kidney disease, Atrial fibrillation and flutter, Aortic aneurysm, Lower extremity peripheral arterial disease.

**Table 2:** The ASDR and ASYR of major diseases caused by high-sodium diets in 2021.

**Disease**	**ASDR**		**ASYR**	
	**1990**	**2021**	**1990**	**2021**
Stroke	14.83	8.70	343.12	200.37
Ischemic heart disease	10.38	7.86	213.88	163.37
Hypertensive heart disease	5.90	3.70	121.71	70.13
Stomach cancer	1.74	0.89	44.53	20.78
Chronic kidney disease	0.73	0.84	18.55	19.81
Atrial fibrillation and flutter	0.12	0.12	3.23	3.36
Aortic aneurysm	0.02	0.02	0.37	0.34
Lower extremity peripheral arterial disease	0.01	0.01	0.16	0.13

### 3.6 Future forecasts of the global burden of high-sodium diets

**Figure 5:**
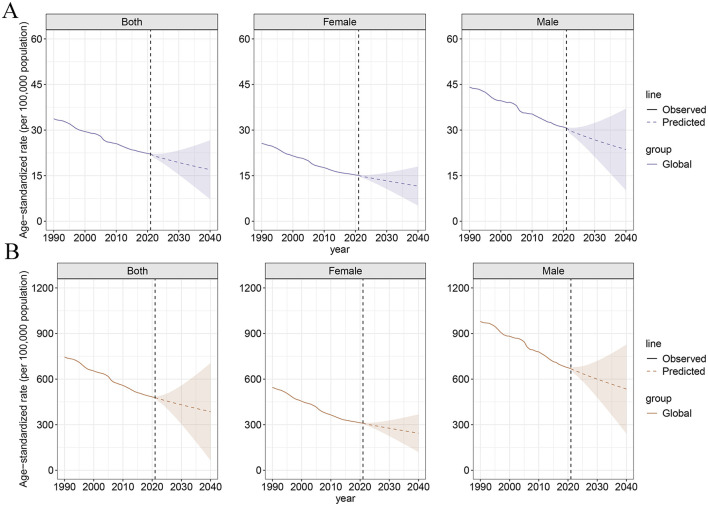
The BAPC model's prediction of ASDR **(A)** and ASYR **(B)** attributable to high-sodium diets by different genders from 2022 to 2040.

Figure 5 shows the projected ASDR and ASYR attributable to high-sodium diets by gender from 2022 to 2040. As shown, both ASDR and ASYR are expected to decline in both males and females over this period. Globally, ASDR is projected to decrease from 22.12 per 100,000 population in 2021 to 16.97 per 100,000 population in 2040—a reduction of 23.28%. Similarly, ASYR is projected to decline from 478.29 to 385.21 per 100,000 population, representing a 19.46% decrease. Among females, the ASDR is expected to fall from 15.11 to 11.58 per 100,000 population (a 23.36% decrease), while ASYR is expected to decline from 310.48 to 243.88 per 100,000 population (a 21.45% decrease). For males, ASDR is anticipated to decrease from 30.70 to 23.62 per 100,000 population (a 23.09% reduction), and ASYR from 667.98 to 534.53 per 100,000 population (a 19.98% reduction) by 2040 (Supplementary Table S4).

## 4 Discussion

High-sodium diets have been linked to numerous health conditions, including hypertension, cardiovascular diseases, gastric cancer, and chronic kidney disease. Despite their significant impact, only one prior study has specifically ed the disease burden attributable to high-sodium diets (11). This study focuses on regional differences and examines the impact of population growth and aging on sodium-related mortality rates, highlighting its significant research value. However, that study did not examine differences across gender and age groups or explore specific disease types associated with high sodium intake (11). In addition, the 2023 WHO Salt Reduction report focuses on the salt intake levels across WHO member states and the changes in sodium intake resulting from policy intervention. In contrast, our study primarily emphasizes the disease burden associated with high-sodium diets. Based on this, we conducted a comprehensive global and regional analysis using data from the GBD 2021, covering the period from 1990 to 2021. This study encompasses 204 countries or regions globally and spans nearly three decades. By identifying the populations and areas most affected by high-sodium diets, our findings aim to inform targeted interventions and guide effective public health strategies.

Our findings show that the global ASDR and ASYR attributable to high-sodium diets declined trend from 1990 to 2021. In addition, the BAPC model predicted that the disease burden of high-sodium diets also showed a downward trend from 2022 to 2040. This may be primarily attributed to the following two reasons. Firstly, over the past three decades, there has been significant improvement in the prevention and treatment of diseases related to high sodium intake, such as cardiovascular diseases, stomach cancer, and chronic kidney disease (11). Second, this trend may be attributed to the implementation of salt-reduction policies. These policies primarily achieve effective population-level sodium intake control through establishing food sodium standards, strengthening nutrition and health education, and implementing nutrition labeling regulations (18). This study also found that the absolute numbers of deaths and disability-adjusted life years caused by high sodium diets are on an upward trend. As previous research has shown, the increase in absolute burden is primarily driven by demographic factors, namely population growth and population aging (11).

From 1990 to 2021, there were marked regional disparities in the disease burden attributable to high-sodium diets, largely reflecting differences in sodium intake levels. Previous studies have demonstrated substantial variations in dietary sodium consumption across regions (10), with East Asia reporting the highest intake. Central Europe and Southeast Asia also exhibit sodium intake levels exceeding the WHO's recommended limits, aligning with the findings of this study regarding countries with a high burden from high-sodium diets. These results underscore the need for targeted sodium reduction strategies in high-burden areas. The Social Development Index (SDI) has been recognized as a key determinant of disease mortality and DALY rates (19). In this study, both the ASDR and ASYR were lowest in high SDI regions, likely due to better access to health education, healthcare services, and coordinated preventive efforts. Interestingly, ASDR and ASYR in middle-SDI regions have now surpassed those in low-middle and low-SDI regions, suggesting a shifting burden and the need for a renewed focus on middle-income settings. Furthermore, the results of this study indicate that at the national level across the 204 countries, the Republic of Bulgaria recorded the highest ASDR and ASYR, while the Islamic Republic of Pakistan experienced the most rapid increases in ASDR and ASYR. Due to data limitations, the specific reasons for these trends remain unclear, which is also one of the main limitations of the research related to GBD. However, previous studies have shown that the sodium intake in the Republic of Bulgaria is 5,089 mg/d (18), significantly higher than the 2,000 mg/d recommended by the WHO. This suggests that the disease burden associated with high-sodium diets in the Republic of Bulgaria is relatively high. Additionally, two other studies have shown an increasing trend in sodium intake in several South Asian countries, which may help explain the rising ASDR and ASYR in the Islamic Republic of Pakistan (3, 18). Nevertheless, the reasons for the fastest increase in ASDR and ASYR due to high-sodium diets in the Islamic Republic of Pakistan remain unknown, and more detailed studies are still needed to explore the underly causes.

Our analysis revealed that the disease burden attributable to high-sodium diets is higher in men than in women, which is consistent with previous findings (11). Men tend to excrete more sodium in their urine (247 mmol/d) compared to women (218 mmol/d) (20), suggesting a higher internal exposure to sodium and potentially greater health risks. Moreover, men are generally more likely to engage in unhealthy behaviors, such as smoking and excessive alcohol consumption (21), both of which are associated with a preference for foods and increased sodium intake. Physiologically, women are less susceptible to the adverse effects of high sodium intake, as they appear to regulate sodium balance more effectively under high-salt dietary conditions (22). These differences underscore the importance of developing gender-based sodium reduction strategies. Additionally, this study also found that both the ASDR and ASYR are attributable to high-sodium diets, which increase progressively with age. Therefore, public health intervention should prioritize education on appropriate sodium intake and promote a low-sodium diet—particularly among older adults and populations with a strong preference for salty foods.

The results of this study indicate that the top five disease burdens attributable to high sodium diets are stroke, Ischemic heart disease, hypertensive heart disease, stomach cancer, and chronic kidney disease. Previous studies have explored the correlation between high sodium intake and conditions such as stroke, hypertension, and ischemic heart disease (23–25). The underlying mechanism through which high-sodium diets contribute to these diseases involves disruption of the renin-angiotensin-aldosterone system and an increase in cardiac output, both of which lead to elevated blood pressure. Sustainable increases in blood pressure and cardiac output subsequently contribute to the development and progression of cardiovascular diseases (26). In addition, high sodium consumption is associated with gastric cancer (5). Excessive salt intake may facilitate colonization by Helicobacter pylori in the stomach, a well-established risk factor for gastric cancer (27). High-sodium diets may also promote cancer progression through various mechanisms, including immune modulation, alternations in gut microbiota, and increased inflammation (28). While the pathophysiological connection between sodium intake and the development of chronic kidney disease is not yet fully understood, several plausible mechanisms have been proposed. Some studies suggest that high salt intake may lead to greater consumption of sugar-sweetened beverages, obesity, and hypertension—all known risk factors for chronic kidney disease (29–32). Furthermore, animal studies have demonstrated that high-sodium diets can exacerbate to exacerbate renal fibrosis and disrupt fatty acid metabolism (33).

This study has several limitations. First, the accuracy of the estimates may be influenced by the quality and availability of data sources across different countries and regions. In particular, the lack of reliable epidemiological data may result in an underestimation of the true disease burden. Second, the GBD methodology relies on various assumptions and modeling techniques, which may introduce a degree of uncertainty into the estimates. Compared to the high-sodium diet data from 2019, the GBD collaborators did not update the exposure data sources used in the dietary risk factor model in 2021, which may also have an impact on our research findings. Third, due to limitations in data collection, it is challenging to thoroughly investigate the reasons behind the regional disparities in disease burden.

## 5 Conclusions

Between 1990 and 2021, both the ASDR and ASYR attributable to high-sodium diets showed a declining trend. Projections from 2021 to 2040 indicate further reductions, with ASDR expected to decrease by 23.28% and ASYR by 19.46%. Regions such as Central Europe, East Asia, and Southeast Asia bear the highest disease burden. Males and older adults are disproportionately affected by high-sodium diets, which are linked to several health conditions, including cardiovascular diseases, gastric cancer, diabetes, and chronic kidney disease. It is necessary to implement health education, nutrition labeling, and other salt reduction-related measures targeting high-risk groups and regions in order to mitigate the disease burden caused by high-sodium diets.

## Data Availability

The datasets presented in this study can be found in online repositories. The names of the repository/repositories and accession number(s) can be found below: https://vizhub.healthdata.org/gbd-results/.
