# Supplementary material for: Global burden of disease from high-sodium diets, 1990–2021: analysis of GBD 2021 data
Source: Front Nutr. 2025 Jul 2;12:1617644. doi: 10.3389/fnut.2025.1617644 (PMC12263369; doi:10.3389/fnut.2025.1617644)
Supplement: Supplementary file 1 [file Table_1.docx]

Supplementary Material

# Supplementary Tables

**Supplementary Table 1.** The number and age-standardized rate of death of high-sodium diets in 1990 and 2021 for all regions

|  | location | Number in 1990 | ASDR in 1990 | Number in 2021 | ASDR in 2021 |
| --- | --- | --- | --- | --- | --- |
| 1 | Andean Latin America | 3640.7 (139.4-10062.9) | 20.1 (0.8-55) | 6744 (186.8-19610.6) | 11.9 (0.3-34.6) |
| 2 | Australasia | 1427 (4.7-6168.7) | 6.3 (0-26.9) | 1322.2 (2.9-5734.9) | 2.2 (0-9.5) |
| 3 | Caribbean | 4384.7 (46.9-13909) | 19 (0.2-59.5) | 6644.7 (55.9-21789.2) | 12.1 (0.1-40) |
| 4 | Central Asia | 21844.3 (2958.3-50384.8) | 52.7 (7.1-121.6) | 20749.7 (1309.6-54916.6) | 32.3 (2.1-83) |
| 5 | Central Europe | 118347.8 (39999.5-211934.4) | 87 (28.7-156.4) | 106504 (30157.2-200313.9) | 44.6 (12.6-84) |
| 6 | Central Latin America | 15659.2 (1733-39990.6) | 22.2 (2.4-56.8) | 36246.4 (3367.5-95530.1) | 15.2 (1.4-40) |
| 7 | Central Sub-Saharan Africa | 1691.7 (0.2-8397.2) | 10.9 (0-51) | 4036 (1.9-19192.1) | 10.9 (0-49.3) |
| 8 | East Asia | 534405.1 (194202.9-980516.9) | 74.9 (23.8-140.2) | 873570.1 (251374.2-1742140.3) | 43 (11.3-87.4) |
| 9 | Eastern Europe | 70406 (3081.4-220176.2) | 27.1 (1.1-85.1) | 69105.9 (2795.6-217939.6) | 19.4 (0.9-60.8) |
| 10 | Eastern Sub-Saharan Africa | 26194.8 (2967.8-57636.8) | 44.8 (4.8-96.7) | 33747.6 (2059.8-83829.6) | 27.1 (1.7-65.8) |
| 11 | Global | 1221009.8 (310504-2716779.2) | 33.7 (8-76.7) | 1857695.6 (367760.9-4251577.1) | 22.1 (4.3-50.9) |
| 12 | High SDI | 197539.3 (29612.5-528416.8) | 17.7 (2.6-47.7) | 206804 (23080.5-554443.7) | 8.7 (1-23.2) |
| 13 | High-income Asia Pacific | 57052.5 (12615.5-121816.5) | 31.1 (6.5-66.9) | 51172.3 (6033.6-124240.9) | 8.4 (1-20.8) |
| 14 | High-income North America | 30895.8 (92.6-118778) | 8.5 (0-32.6) | 51605.5 (1079.9-163446.9) | 7.6 (0.2-23.5) |
| 15 | High-middle SDI | 394153.9 (111044.1-857090.5) | 43.5 (11.6-96.9) | 548279.3 (130100.8-1196037.5) | 28 (6.5-61.5) |
| 16 | Low SDI | 43613.2 (3046.9-119067.3) | 23.7 (1.6-65.2) | 72326.4 (2697.3-211391.2) | 18 (0.6-52.1) |
| 17 | Low-middle SDI | 128433.9 (19542.7-320837.8) | 24.2 (3.3-62) | 262824.1 (26468.1-693409.8) | 20.4 (1.9-54.6) |
| 18 | Middle SDI | 454900.1 (142763.8-893020.6) | 51.4 (14.3-102.2) | 765167.8 (183288.4-1653813) | 31 (6.8-67.9) |
| 19 | North Africa and Middle East | 14401.7 (4.8-70537.2) | 9.7 (0-50.1) | 25791.8 (11.8-135913.3) | 6.5 (0-35.5) |
| 20 | Oceania | 956.7 (153-2284.1) | 45.8 (7.5-104.3) | 2116.2 (263.4-4939.4) | 37.1 (4.5-85) |
| 21 | South Asia | 81961.5 (2892.2-240510.7) | 16.3 (0.5-49) | 218639.3 (10594.2-617060.9) | 16.2 (0.7-46.1) |
| 22 | Southeast Asia | 127914.3 (37969.5-239220.8) | 56.7 (15.4-107.7) | 229839.2 (43789-474677.6) | 38.8 (6.8-81.7) |
| 23 | Southern Latin America | 9998.9 (274.5-27187.1) | 23.5 (0.7-63.7) | 10135.6 (260.1-27476.2) | 11.1 (0.3-30.2) |
| 24 | Southern Sub-Saharan Africa | 2185.8 (6.1-7972.9) | 8.9 (0-33.8) | 4222.8 (4.4-17476.7) | 8.6 (0-36.1) |
| 25 | Tropical Latin America | 21541.5 (1173.5-56136.7) | 27.7 (1.5-72.7) | 30507 (1326.9-83630.7) | 12.2 (0.5-33.5) |
| 26 | Western Europe | 65954.9 (1128.4-229897.3) | 11.1 (0.2-38.7) | 54927 (735.7-188212.8) | 4.8 (0.1-16.2) |
| 27 | Western Sub-Saharan Africa | 10144.6 (15.7-37785) | 13.9 (0-52.5) | 20068.3 (60.6-69273.1) | 12.9 (0-44.9) |

**Supplementary Table 2.** The number and age-standardized rate of DALYs of high-sodium diets in 1990 and 2021 for all regions

|  | location | Number in 1990 | ASYR in 1990 | Number in 2021 | ASYR in 2021 |
| --- | --- | --- | --- | --- | --- |
| 1 | Andean Latin America | 78581.7 (2925.7-223454.7) | 396.5 (15.1-1113) | 137092.1 (4029-410606.6) | 234.4 (6.9-697.2) |
| 2 | Australasia | 30542.8 (169.8-125482.7) | 132.7 (0.8-539.1) | 24777.7 (106.9-101093.6) | 47.8 (0.3-189.4) |
| 3 | Caribbean | 86495.7 (769-284875) | 347.4 (3.4-1131.3) | 130569 (864.1-449743.6) | 241.7 (1.6-834.1) |
| 4 | Central Asia | 462082.1 (60460.3-1095640.1) | 1022.1 (134.6-2394.1) | 422720.7 (24832.1-1153563.3) | 571.4 (33.7-1533.4) |
| 5 | Central Europe | 2384611.3 (831256.3-4304214.3) | 1646.1 (567.9-2976.1) | 1812979.7 (524986.4-3390298.5) | 797.1 (231.8-1500.4) |
| 6 | Central Latin America | 351097.7 (41110-892183.2) | 439.4 (50.5-1111.3) | 760959.1 (73019-1993103.6) | 306.5 (29.4-801) |
| 7 | Central Sub-Saharan Africa | 43237 (5.6-217817.9) | 214 (0-1041.7) | 98121.2 (33.5-470598.9) | 204 (0.1-964) |
| 8 | East Asia | 13533882.1 (5291686.3-24876943.2) | 1597.5 (583-2951.9) | 19325695 (6641344.7-36588126.4) | 890.6 (294.1-1700.5) |
| 9 | Eastern Europe | 1652760.8 (99372.9-4862223.3) | 599.2 (34.8-1774.1) | 1498643.5 (85803.8-4409523.6) | 434.4 (26.8-1268) |
| 10 | Eastern Sub-Saharan Africa | 639561.2 (69637-1441955.5) | 919.2 (101.7-2026.2) | 760156.9 (43320.5-1973839.1) | 514.2 (31-1280.3) |
| 11 | Global | 29378002.2 (8128904.9-63898036.7) | 745.5 (199.4-1624.2) | 41275914.1 (9297839.4-91456678.5) | 478.3 (105.9-1064.9) |
| 12 | High SDI | 4009659.9 (670881.7-10485734.2) | 364.4 (61.4-953) | 3854370.4 (474387.2-9909951.8) | 185 (24.1-468.5) |
| 13 | High-income Asia Pacific | 1242731 (295413.6-2625973) | 631.2 (145.8-1340.2) | 863716.8 (103586-2055019.4) | 178.7 (22.6-429.3) |
| 14 | High-income North America | 601395 (2308-2238682.1) | 172.1 (0.7-641.3) | 1094375.2 (32091.9-3210737.9) | 176.4 (6-499) |
| 15 | High-middle SDI | 9318540.6 (2870896.6-19557331.9) | 948.4 (282.9-2004.5) | 11594648.9 (3270627.2-24107173.4) | 587.6 (166.3-1227.8) |
| 16 | Low SDI | 1101926.9 (74445.1-3066578.6) | 506.2 (34.5-1390.1) | 1748609.1 (65744.8-5146317.4) | 364.9 (14-1062.3) |
| 17 | Low-middle SDI | 3382563.6 (553029.9-8436568.6) | 551 (84.7-1371.9) | 6507258 (718585.3-16709882.7) | 451.9 (47.8-1171.3) |
| 18 | Middle SDI | 11516271.1 (3904472.6-22722960.6) | 1126.1 (359.1-2215.8) | 17527988 (4731094.4-36207853.9) | 654.8 (168.3-1371.9) |
| 19 | North Africa and Middle East | 371113.4 (237.1-1742101.9) | 216.6 (0.1-1048.2) | 644597.6 (543.6-3220389.5) | 139.7 (0.1-721.5) |
| 20 | Oceania | 22293.9 (3180.3-57948.5) | 887.9 (137.4-2135.4) | 50672.4 (5679.2-124563.8) | 754.6 (94-1768.3) |
| 21 | South Asia | 2259618.8 (99173.7-6447253.9) | 381.8 (14.5-1104.2) | 5659390.2 (343279.9-15462108.8) | 376.4 (21.2-1038.1) |
| 22 | Southeast Asia | 3341053.5 (1035309.8-6246875) | 1305 (394.8-2439.9) | 5645498.1 (1129766.1-11633280.7) | 856.5 (167-1769.4) |
| 23 | Southern Latin America | 204820.3 (6277.1-561553.8) | 454.2 (14-1242.1) | 188221 (5555.6-512435.4) | 213.7 (6.5-581.9) |
| 24 | Southern Sub-Saharan Africa | 60291.9 (245.9-215141.5) | 212.6 (0.7-768.1) | 106966.9 (179.8-419698.3) | 184.9 (0.2-744.7) |
| 25 | Tropical Latin America | 514149.7 (29827.5-1353717.4) | 574.9 (33-1487.5) | 667596.1 (29883.6-1825086.8) | 260.1 (11.7-710.4) |
| 26 | Western Europe | 1252988.3 (30325.9-4221422.1) | 218.6 (5.7-730.5) | 893702.2 (18745.1-2949385.6) | 92 (2.6-294) |
| 27 | Western Sub-Saharan Africa | 244694.3 (387-902818.5) | 290 (0.5-1075.7) | 489462.6 (1551.1-1708629.1) | 261.3 (0.8-901.6) |

**Supplementary Table 3** The ASDR, ASYR of high-sodium diets in 2021 for all countries, with EAPC from 1990 and 2021

|  | **location** | **ASDR in 2021** | **ASYR in 2021** | **EAPC of ASDR** | **EAPC of ASYR** |
| --- | --- | --- | --- | --- | --- |
| 1 | American Samoa | 17.81 (0.32 to 54.77) | 309.75 (3.91 to 1066.72) | 0.01 (-0.09 to 0.12) | -0.01 (-0.11 to 0.08) |
| 2 | Antigua and Barbuda | 15.83 (0.07 to 50.07) | 275.5 (0.96 to 892.21) | -1.05 (-1.33 to -0.77) | -1.2 (-1.48 to -0.91) |
| 3 | Arab Republic of Egypt | 11.13 (0 to 58.63) | 233.86 (0.04 to 1160.28) | -0.53 (-0.64 to -0.42) | -0.55 (-0.65 to -0.45) |
| 4 | Argentine Republic | 11.71 (0.26 to 31.44) | 221.82 (5.19 to 600.52) | -1.99 (-2.14 to -1.84) | -2.13 (-2.26 to -1.99) |
| 5 | Australia | 1.91 (0 to 8.57) | 41.37 (0.17 to 173.01) | -3.46 (-3.59 to -3.32) | -3.38 (-3.55 to -3.21) |
| 6 | Barbados | 6.56 (0 to 26.58) | 124.58 (0.07 to 509.84) | -1.44 (-1.62 to -1.25) | -1.4 (-1.57 to -1.22) |
| 7 | Belize | 11.51 (0.06 to 36.28) | 220.89 (1.07 to 715.98) | -0.91 (-1.3 to -0.53) | -0.87 (-1.22 to -0.51) |
| 8 | Bermuda | 7.03 (0.04 to 22.3) | 124.94 (0.72 to 412.54) | -3.32 (-3.62 to -3.01) | -3.18 (-3.51 to -2.84) |
| 9 | Bolivarian Republic of Venezuela | 23.4 (1.67 to 59.8) | 487.31 (38.53 to 1232.1) | -1.08 (-1.33 to -0.84) | -1.21 (-1.45 to -0.96) |
| 10 | Bosnia and Herzegovina | 46.42 (12.73 to 87.74) | 832.57 (231.18 to 1580) | -1.8 (-2.07 to -1.54) | -2.15 (-2.4 to -1.9) |
| 11 | Brunei Darussalam | 24.81 (3.22 to 54.12) | 497.58 (74.11 to 1084.22) | -1.86 (-2.11 to -1.61) | -2.24 (-2.46 to -2.02) |
| 12 | Burkina Faso | 14.53 (0.01 to 53.54) | 294.68 (0.28 to 1075.55) | 0.79 (0.66 to 0.92) | 0.6 (0.5 to 0.69) |
| 13 | Canada | 4.64 (0.07 to 14.6) | 100.87 (2.89 to 303.85) | -2.95 (-3.07 to -2.83) | -2.58 (-2.71 to -2.44) |
| 14 | Central African Republic | 20.19 (0.02 to 78.14) | 378.84 (0.26 to 1568.1) | 0.16 (0 to 0.32) | 0.05 (-0.1 to 0.2) |
| 15 | Commonwealth of Dominica | 18.75 (0.08 to 61.24) | 338.8 (1.37 to 1125.51) | -1.39 (-1.57 to -1.2) | -1.08 (-1.29 to -0.88) |
| 16 | Commonwealth of the Bahamas | 16.7 (0.08 to 53.12) | 328.41 (1.39 to 1095.34) | -0.82 (-1 to -0.64) | -0.79 (-0.92 to -0.66) |
| 17 | Cook Islands | 34.1 (3.93 to 71.72) | 654.96 (72.5 to 1429.64) | -2.52 (-2.65 to -2.39) | -2.33 (-2.45 to -2.2) |
| 18 | Czech Republic | 31.43 (9.17 to 58.68) | 552.53 (165.79 to 1020.69) | -3.54 (-3.64 to -3.43) | -3.88 (-4 to -3.76) |
| 19 | Democratic People's Republic of Korea | 56.47 (13.63 to 112.41) | 1240.17 (336.42 to 2465.23) | -0.61 (-0.74 to -0.48) | -0.76 (-0.91 to -0.61) |
| 20 | Democratic Republic of Sao Tome and Principe | 13.39 (0.01 to 49.04) | 275.15 (0.22 to 995.05) | 0.7 (0.55 to 0.85) | 0.49 (0.32 to 0.67) |
| 21 | Democratic Republic of Timor-Leste | 50.17 (7.85 to 109.73) | 1054.07 (177.82 to 2299.17) | -0.62 (-0.83 to -0.42) | -0.88 (-1.09 to -0.67) |
| 22 | Democratic Republic of the Congo | 7.94 (0 to 42.43) | 154.09 (0 to 817.2) | 0.05 (-0.01 to 0.11) | -0.15 (-0.21 to -0.1) |
| 23 | Democratic Socialist Republic of Sri Lanka | 28.65 (3.68 to 64.14) | 575.21 (82.5 to 1244.12) | -2.09 (-2.24 to -1.95) | -2.37 (-2.53 to -2.2) |
| 24 | Dominican Republic | 13.38 (0.09 to 44.5) | 264.58 (1.27 to 910.25) | -0.29 (-0.51 to -0.07) | 0.05 (-0.11 to 0.21) |
| 25 | Eastern Republic of Uruguay | 12.26 (0.4 to 33.36) | 237.56 (9.14 to 643.78) | -2.32 (-2.43 to -2.2) | -2.46 (-2.58 to -2.34) |
| 26 | Federal Democratic Republic of Ethiopia | 21.66 (1.3 to 52.38) | 407.35 (23.55 to 1019.33) | -3.39 (-3.59 to -3.19) | -3.78 (-4 to -3.57) |
| 27 | Federal Democratic Republic of Nepal | 19.54 (0.28 to 53.9) | 374.92 (4.98 to 1062.91) | 1.48 (1.19 to 1.78) | 1.26 (0.96 to 1.56) |
| 28 | Federal Republic of Germany | 6.08 (0.07 to 20.07) | 120.97 (2.37 to 377.26) | -2.49 (-2.62 to -2.36) | -2.62 (-2.78 to -2.46) |
| 29 | Federal Republic of Nigeria | 9.43 (0.01 to 35.16) | 182.96 (0.28 to 682.26) | -1.22 (-1.35 to -1.08) | -1.31 (-1.45 to -1.17) |
| 30 | Federal Republic of Somalia | 32.61 (1.19 to 86.06) | 650.85 (24.14 to 1743.44) | -1.79 (-1.88 to -1.71) | -1.98 (-2.08 to -1.88) |
| 31 | Federated States of Micronesia | 53.07 (5.97 to 124.23) | 1072.9 (127.74 to 2628.45) | -0.89 (-0.94 to -0.84) | -0.77 (-0.82 to -0.72) |
| 32 | Federative Republic of Brazil | 12.15 (0.52 to 33.3) | 258.61 (11.35 to 710.22) | -2.64 (-2.7 to -2.59) | -2.66 (-2.73 to -2.6) |
| 33 | French Republic | 2.95 (0.01 to 10.58) | 57.3 (0.23 to 204.12) | -2.58 (-2.65 to -2.51) | -2.21 (-2.28 to -2.14) |
| 34 | Gabonese Republic | 17.45 (0.02 to 64.12) | 298.76 (0.22 to 1151.56) | -0.02 (-0.26 to 0.22) | -0.18 (-0.41 to 0.04) |
| 35 | Georgia | 30.8 (2 to 78.18) | 557.22 (31.1 to 1439.85) | -1.93 (-2.11 to -1.74) | -2.37 (-2.53 to -2.2) |
| 36 | Grand Duchy of Luxembourg | 4.17 (0.02 to 14.66) | 78.68 (0.7 to 256.74) | -3.38 (-3.51 to -3.26) | -3.5 (-3.62 to -3.39) |
| 37 | Greenland | 9.05 (0.1 to 29.54) | 206.54 (3.17 to 652.6) | -2.14 (-2.22 to -2.05) | -2.05 (-2.1 to -2) |
| 38 | Grenada | 15.17 (0.05 to 49.64) | 287.86 (1.07 to 938.93) | -1.32 (-1.55 to -1.09) | -1.31 (-1.46 to -1.15) |
| 39 | Guam | 17.95 (2.1 to 41.3) | 427.16 (45.69 to 1022.87) | -2.77 (-3.21 to -2.32) | -1.97 (-2.34 to -1.6) |
| 40 | Hashemite Kingdom of Jordan | 4.31 (0 to 23.36) | 92.12 (0.01 to 457.01) | -2.3 (-2.48 to -2.11) | -2.46 (-2.66 to -2.26) |
| 41 | Hellenic Republic | 7.38 (0.05 to 25.16) | 145.21 (1.77 to 469.78) | -2.15 (-2.25 to -2.05) | -1.82 (-1.89 to -1.76) |
| 42 | Hungary | 46.68 (15.63 to 82.28) | 907.85 (336.77 to 1566.69) | -2.36 (-2.46 to -2.26) | -2.75 (-2.86 to -2.63) |
| 43 | Independent State of Papua New Guinea | 36.78 (4.2 to 85.63) | 742.5 (86.43 to 1757.38) | -0.66 (-0.73 to -0.59) | -0.6 (-0.69 to -0.51) |
| 44 | Independent State of Samoa | 19.66 (0.28 to 63.22) | 306.59 (3.2 to 1118.29) | 0.24 (0.07 to 0.41) | 0.24 (0.09 to 0.39) |
| 45 | Ireland | 2.82 (0 to 11.2) | 56.31 (0.12 to 215.03) | -4.19 (-4.34 to -4.04) | -4.32 (-4.47 to -4.17) |
| 46 | Islamic Republic of Afghanistan | 12.39 (0 to 67.15) | 282.93 (0.04 to 1439.44) | -1.47 (-1.65 to -1.29) | -1.58 (-1.77 to -1.39) |
| 47 | Islamic Republic of Iran | 5.05 (0 to 26.33) | 106.09 (0.08 to 518.74) | -1.83 (-1.92 to -1.74) | -1.97 (-2.05 to -1.88) |
| 48 | Islamic Republic of Mauritania | 14.42 (0.01 to 52.14) | 286.35 (0.32 to 1037.85) | -0.72 (-0.83 to -0.62) | -0.95 (-1.06 to -0.84) |
| 49 | Islamic Republic of Pakistan | 22.28 (0.46 to 63.87) | 455.6 (8.96 to 1305.86) | 1.76 (1.52 to 1.99) | 1.78 (1.54 to 2.02) |
| 50 | Jamaica | 12.95 (0.08 to 41.19) | 243.65 (1.16 to 794.46) | -1.05 (-1.52 to -0.58) | -0.84 (-1.3 to -0.38) |
| 51 | Japan | 7.48 (0.62 to 19.36) | 162.33 (14.47 to 408.85) | -4.57 (-4.81 to -4.33) | -4.43 (-4.65 to -4.22) |
| 52 | Kingdom of Bahrain | 4.88 (0 to 26.31) | 95.07 (0.02 to 479.03) | -3.1 (-3.45 to -2.75) | -3.37 (-3.69 to -3.05) |
| 53 | Kingdom of Belgium | 5.09 (0.09 to 14.92) | 95.59 (1.81 to 276.34) | -3.73 (-3.82 to -3.65) | -3.65 (-3.72 to -3.58) |
| 54 | Kingdom of Bhutan | 15.88 (0.18 to 45.16) | 303.53 (4.23 to 876.81) | 0.99 (0.84 to 1.15) | 0.74 (0.58 to 0.91) |
| 55 | Kingdom of Cambodia | 45.97 (6.71 to 99.06) | 944.01 (149.63 to 2010.4) | -1.63 (-1.77 to -1.48) | -2.04 (-2.18 to -1.9) |
| 56 | Kingdom of Denmark | 4.06 (0.02 to 13.67) | 80.76 (0.66 to 259.91) | -3.95 (-4.1 to -3.8) | -3.93 (-4.08 to -3.77) |
| 57 | Kingdom of Eswatini | 12.24 (0 to 54.92) | 280.13 (0.16 to 1178.15) | -0.3 (-0.74 to 0.13) | -0.25 (-0.75 to 0.25) |
| 58 | Kingdom of Lesotho | 15.12 (0 to 66.83) | 337.64 (0.21 to 1442.89) | 1.55 (1.11 to 1.98) | 1.63 (1.19 to 2.08) |
| 59 | Kingdom of Morocco | 7.64 (0 to 41.17) | 162.05 (0.02 to 823.36) | -0.45 (-0.56 to -0.35) | -0.7 (-0.79 to -0.6) |
| 60 | Kingdom of Norway | 2.89 (0.02 to 10.48) | 57.79 (0.56 to 199.72) | -4.22 (-4.31 to -4.12) | -4.33 (-4.42 to -4.25) |
| 61 | Kingdom of Saudi Arabia | 7.58 (0 to 40.3) | 173.38 (0.03 to 865.03) | -0.99 (-1.13 to -0.85) | -0.78 (-0.93 to -0.64) |
| 62 | Kingdom of Spain | 2.08 (0.02 to 9.4) | 52.84 (0.77 to 202.43) | -2.3 (-2.42 to -2.17) | -1.78 (-1.9 to -1.67) |
| 63 | Kingdom of Sweden | 4.58 (0.03 to 15.33) | 83.85 (0.76 to 269.07) | -2.86 (-2.92 to -2.81) | -3.06 (-3.12 to -3) |
| 64 | Kingdom of Thailand | 15.94 (2.22 to 36.23) | 366.54 (57.48 to 828.93) | -2.99 (-3.22 to -2.76) | -2.92 (-3.15 to -2.68) |
| 65 | Kingdom of Tonga | 24.12 (2.45 to 57.15) | 483.68 (49.69 to 1165.08) | -0.48 (-0.64 to -0.32) | -0.42 (-0.59 to -0.25) |
| 66 | Kingdom of the Netherlands | 3.46 (0.01 to 12.69) | 64.12 (0.47 to 223.77) | -2.74 (-2.9 to -2.58) | -2.97 (-3.13 to -2.82) |
| 67 | Kyrgyz Republic | 30.24 (1.7 to 81.82) | 539.62 (27.88 to 1543.93) | -1.74 (-1.94 to -1.55) | -2.23 (-2.46 to -1.99) |
| 68 | Lao People's Democratic Republic | 54.76 (7.88 to 118.2) | 1163.25 (184.96 to 2488.4) | -2.36 (-2.5 to -2.22) | -2.73 (-2.87 to -2.58) |
| 69 | Lebanese Republic | 2.91 (0 to 15.38) | 63.02 (0.01 to 305.07) | -3.22 (-3.45 to -2.99) | -3.3 (-3.58 to -3.01) |
| 70 | Malaysia | 32.36 (5.11 to 69.29) | 718.94 (122.39 to 1504.27) | -1.84 (-1.96 to -1.71) | -1.99 (-2.13 to -1.85) |
| 71 | Mongolia | 30.87 (1.67 to 84.8) | 584.66 (30.79 to 1677.96) | -2.81 (-3.06 to -2.56) | -2.83 (-3.08 to -2.57) |
| 72 | Montenegro | 76.37 (21.7 to 139.98) | 1235.43 (380.26 to 2241.35) | 0.27 (-0.01 to 0.56) | -0.44 (-0.61 to -0.27) |
| 73 | New Zealand | 4.03 (0.02 to 14.68) | 81.77 (0.54 to 282.99) | -3.32 (-3.42 to -3.22) | -3.48 (-3.63 to -3.33) |
| 74 | North Macedonia | 91.88 (26.02 to 169.06) | 1433.91 (401.31 to 2659.94) | -0.84 (-1.23 to -0.46) | -1.5 (-1.76 to -1.24) |
| 75 | Northern Mariana Islands | 27.23 (2.79 to 63.43) | 541.61 (57.36 to 1291.13) | -0.59 (-0.73 to -0.44) | -0.43 (-0.54 to -0.31) |
| 76 | Palestine | 6.22 (0 to 34.33) | 125.15 (0.01 to 637.04) | -1.83 (-2.07 to -1.59) | -1.84 (-2.04 to -1.64) |
| 77 | People's Democratic Republic of Algeria | 6.22 (0 to 37.02) | 116.68 (0.01 to 630.37) | -1.19 (-1.28 to -1.1) | -1.47 (-1.54 to -1.41) |
| 78 | People's Republic of Bangladesh | 20.25 (0.25 to 58.35) | 384.91 (4.79 to 1114.45) | 0.73 (0.32 to 1.14) | 0.6 (0.28 to 0.92) |
| 79 | People's Republic of China | 43.5 (11.5 to 88.8) | 899.57 (297.94 to 1718.1) | -1.77 (-1.85 to -1.68) | -1.89 (-1.96 to -1.81) |
| 80 | Plurinational State of Bolivia | 18.75 (0.36 to 56.85) | 355.24 (7.48 to 1093.87) | -1.58 (-1.69 to -1.47) | -1.78 (-1.91 to -1.66) |
| 81 | Portuguese Republic | 4.68 (0.07 to 16.91) | 105.79 (2.96 to 349.65) | -2.69 (-2.8 to -2.59) | -2.51 (-2.62 to -2.41) |
| 82 | Principality of Andorra | 2.88 (0 to 11.92) | 56.82 (0.14 to 225.35) | -1.76 (-2.03 to -1.49) | -1.83 (-2.08 to -1.58) |
| 83 | Principality of Monaco | 5.25 (0.04 to 18.69) | 103.13 (1.22 to 345.31) | -1.95 (-2.05 to -1.84) | -1.9 (-2.01 to -1.8) |
| 84 | Puerto Rico | 6.1 (0.04 to 19.52) | 119.61 (0.62 to 393.04) | -3.09 (-3.29 to -2.89) | -2.68 (-2.87 to -2.49) |
| 85 | Republic of Albania | 54.12 (15.62 to 102.45) | 862.52 (249.42 to 1649.41) | -0.94 (-1.17 to -0.71) | -1.41 (-1.64 to -1.18) |
| 86 | Republic of Angola | 16.78 (0.02 to 63.4) | 295.49 (0.19 to 1177.38) | -0.21 (-0.36 to -0.05) | -0.45 (-0.58 to -0.32) |
| 87 | Republic of Armenia | 24.35 (1.38 to 64.16) | 419.13 (21.98 to 1142.57) | -3 (-3.21 to -2.78) | -3.06 (-3.26 to -2.87) |
| 88 | Republic of Austria | 9.83 (0.36 to 26.2) | 166.15 (6.11 to 443.35) | -2.08 (-2.22 to -1.93) | -2.34 (-2.51 to -2.17) |
| 89 | Republic of Azerbaijan | 33.59 (1.9 to 88.81) | 573.56 (28.65 to 1576.62) | -1.42 (-1.62 to -1.23) | -2.04 (-2.2 to -1.88) |
| 90 | Republic of Belarus | 13.05 (0.02 to 54.39) | 281.95 (0.56 to 1103.83) | -1.19 (-1.54 to -0.84) | -1.33 (-1.75 to -0.9) |
| 91 | Republic of Benin | 15.67 (0.05 to 50.79) | 310.72 (0.92 to 1036.12) | -0.2 (-0.27 to -0.13) | -0.38 (-0.46 to -0.3) |
| 92 | Republic of Botswana | 8.15 (0 to 35.39) | 170.85 (0.08 to 717.56) | -1.79 (-2 to -1.59) | -2.05 (-2.25 to -1.85) |
| 93 | Republic of Bulgaria | 103.11 (32.89 to 182.22) | 1831.62 (588.88 to 3263.68) | -1.21 (-1.37 to -1.04) | -1.34 (-1.49 to -1.19) |
| 94 | Republic of Burundi | 26.73 (1.02 to 69.84) | 518.97 (20.76 to 1378.79) | -2.81 (-3.06 to -2.56) | -3.06 (-3.33 to -2.8) |
| 95 | Republic of Cabo Verde | 12.97 (0.01 to 47.98) | 254.21 (0.21 to 946.86) | 0.34 (0.03 to 0.66) | 0.03 (-0.23 to 0.3) |
| 96 | Republic of Cameroon | 14.89 (0.01 to 56.13) | 309.5 (0.31 to 1169.62) | 0.09 (-0.17 to 0.35) | 0.05 (-0.23 to 0.34) |
| 97 | Republic of Chad | 15.72 (0.02 to 58.02) | 331.89 (0.37 to 1224.07) | 0.61 (0.38 to 0.85) | 0.55 (0.31 to 0.79) |
| 98 | Republic of Chile | 9.65 (0.28 to 26.81) | 191.32 (6.01 to 531.43) | -2.42 (-2.49 to -2.34) | -2.44 (-2.5 to -2.37) |
| 99 | Republic of Colombia | 18 (3.61 to 38.37) | 362.73 (77.23 to 782.54) | -2.88 (-3.06 to -2.7) | -2.91 (-3.1 to -2.72) |
| 100 | Republic of Costa Rica | 11.67 (0.7 to 31.68) | 248.27 (16.79 to 664.91) | -2.06 (-2.2 to -1.91) | -1.91 (-2.06 to -1.76) |
| 101 | Republic of Croatia | 37.64 (11.04 to 70.94) | 633.57 (192.47 to 1197.38) | -3.16 (-3.27 to -3.06) | -3.39 (-3.48 to -3.29) |
| 102 | Republic of Cuba | 10.47 (0.06 to 33.3) | 190.43 (0.8 to 613.71) | -1.65 (-1.9 to -1.4) | -1.43 (-1.66 to -1.19) |
| 103 | Republic of Cyprus | 7.9 (0.04 to 27.76) | 129.17 (1.14 to 424.01) | -3.52 (-3.77 to -3.27) | -3.22 (-3.38 to -3.06) |
| 104 | Republic of C么te d'Ivoire | 15.71 (0.02 to 54.88) | 321.95 (0.53 to 1153.65) | -0.18 (-0.39 to 0.03) | -0.29 (-0.51 to -0.08) |
| 105 | Republic of Djibouti | 27.13 (0.99 to 72.56) | 512.05 (19.15 to 1398.29) | -1.84 (-1.94 to -1.74) | -2.02 (-2.12 to -1.91) |
| 106 | Republic of Ecuador | 14.92 (0.41 to 43.16) | 269.87 (8.17 to 806.77) | -1.07 (-1.35 to -0.79) | -1.27 (-1.54 to -0.99) |
| 107 | Republic of El Salvador | 16.13 (1.03 to 44.02) | 345.35 (23.17 to 940.55) | -0.54 (-0.72 to -0.35) | -0.55 (-0.76 to -0.35) |
| 108 | Republic of Equatorial Guinea | 14.93 (0.03 to 57.94) | 257.17 (0.37 to 1034.2) | -0.92 (-1.11 to -0.72) | -1.31 (-1.54 to -1.07) |
| 109 | Republic of Estonia | 6.53 (0 to 30.37) | 123.22 (0.02 to 551.17) | -1.8 (-1.96 to -1.63) | -2.39 (-2.58 to -2.19) |
| 110 | Republic of Fiji | 44.35 (5.05 to 101.31) | 874.07 (100.34 to 2051.84) | -1.08 (-1.28 to -0.87) | -0.98 (-1.17 to -0.8) |
| 111 | Republic of Finland | 6.22 (0.07 to 20.45) | 124.25 (2.27 to 384.06) | -2.48 (-2.63 to -2.32) | -2.8 (-2.94 to -2.65) |
| 112 | Republic of Ghana | 22.88 (0.19 to 70.94) | 478.19 (4.38 to 1470.6) | 0.65 (0.41 to 0.9) | 0.58 (0.33 to 0.84) |
| 113 | Republic of Guatemala | 16.06 (0.78 to 43.99) | 319.42 (17.65 to 880.1) | -1.13 (-1.34 to -0.92) | -1.07 (-1.29 to -0.84) |
| 114 | Republic of Guinea | 15.29 (0.01 to 56.67) | 320.02 (0.28 to 1184.95) | 0.68 (0.5 to 0.85) | 0.58 (0.41 to 0.75) |
| 115 | Republic of Guinea-Bissau | 21.56 (0.02 to 79.61) | 463.65 (0.53 to 1704.32) | 0.2 (0.09 to 0.31) | -0.01 (-0.12 to 0.1) |
| 116 | Republic of Guyana | 23.39 (0.12 to 74.42) | 464.46 (1.99 to 1514.77) | -1.35 (-1.5 to -1.2) | -1.23 (-1.41 to -1.06) |
| 117 | Republic of Haiti | 23.46 (0.1 to 83.77) | 455.58 (1.37 to 1708.65) | -1.21 (-1.27 to -1.15) | -1.14 (-1.22 to -1.06) |
| 118 | Republic of Honduras | 32.37 (1.87 to 82.07) | 624.24 (37.9 to 1589.97) | 1.17 (0.96 to 1.37) | 0.93 (0.76 to 1.1) |
| 119 | Republic of Iceland | 4.91 (0.07 to 15.61) | 91.87 (1.42 to 284.77) | -2.81 (-2.91 to -2.7) | -3.02 (-3.08 to -2.96) |
| 120 | Republic of India | 15.18 (0.77 to 44.44) | 368.46 (24.22 to 1024.29) | -0.03 (-0.18 to 0.11) | -0.04 (-0.15 to 0.06) |
| 121 | Republic of Indonesia | 49.9 (8.19 to 107.99) | 1079.08 (198.92 to 2271.07) | -0.42 (-0.5 to -0.34) | -0.79 (-0.87 to -0.7) |
| 122 | Republic of Iraq | 7.93 (0 to 42.66) | 171.94 (0.02 to 863.39) | -1.07 (-1.25 to -0.89) | -1.3 (-1.45 to -1.15) |
| 123 | Republic of Italy | 7.12 (0.2 to 22.16) | 128.72 (6.75 to 375.87) | -2.17 (-2.35 to -1.99) | -2.63 (-2.81 to -2.46) |
| 124 | Republic of Kazakhstan | 30.71 (1.79 to 81.71) | 522.64 (27.9 to 1439.47) | -2.58 (-3.02 to -2.15) | -3.17 (-3.68 to -2.67) |
| 125 | Republic of Kenya | 15.88 (0.95 to 42.96) | 283.97 (18.12 to 784.42) | 0.29 (0.12 to 0.46) | 0 (-0.15 to 0.16) |
| 126 | Republic of Kiribati | 40.31 (4.85 to 95.69) | 847.51 (98.02 to 2078.29) | -0.46 (-0.53 to -0.39) | -0.42 (-0.52 to -0.32) |
| 127 | Republic of Korea | 14.31 (2.81 to 29.91) | 255.4 (51.1 to 545.14) | -4.63 (-4.78 to -4.49) | -4.92 (-5.07 to -4.76) |
| 128 | Republic of Latvia | 13.49 (0.22 to 44.3) | 282.91 (5.17 to 887.38) | -2.13 (-2.39 to -1.88) | -2.33 (-2.64 to -2.02) |
| 129 | Republic of Liberia | 15.77 (0.02 to 58.22) | 325.03 (0.32 to 1198.67) | 0.24 (0.12 to 0.36) | 0.15 (0.03 to 0.28) |
| 130 | Republic of Lithuania | 12.95 (0.02 to 44.97) | 240.01 (0.45 to 836.5) | -1.6 (-1.81 to -1.39) | -1.7 (-1.96 to -1.44) |
| 131 | Republic of Madagascar | 41.11 (1.44 to 103.56) | 804.01 (26.42 to 2054.91) | -1.51 (-1.61 to -1.4) | -1.68 (-1.78 to -1.57) |
| 132 | Republic of Malawi | 28.28 (0.95 to 74.23) | 542.71 (19.84 to 1452.38) | -1.22 (-1.43 to -1.01) | -1.44 (-1.67 to -1.21) |
| 133 | Republic of Maldives | 22.72 (3.21 to 48.12) | 447.24 (72.76 to 938.17) | -4.22 (-4.41 to -4.02) | -4.84 (-5.07 to -4.6) |
| 134 | Republic of Mali | 12.01 (0.01 to 44.22) | 255.59 (0.17 to 938.84) | -0.08 (-0.17 to 0) | -0.22 (-0.3 to -0.13) |
| 135 | Republic of Malta | 6.18 (0.17 to 19.39) | 131.31 (6.27 to 379.2) | -2.73 (-2.86 to -2.6) | -2.67 (-2.77 to -2.56) |
| 136 | Republic of Mauritius | 32.33 (4.46 to 69.36) | 702.97 (109.3 to 1471.46) | -3.58 (-3.94 to -3.23) | -3.78 (-4.16 to -3.41) |
| 137 | Republic of Moldova | 11.63 (0.02 to 46.72) | 249.11 (0.51 to 980.07) | -1.98 (-2.21 to -1.75) | -1.6 (-1.88 to -1.32) |
| 138 | Republic of Mozambique | 37.37 (1.29 to 97.59) | 714.79 (23.15 to 1928.57) | -0.35 (-0.46 to -0.25) | -0.44 (-0.55 to -0.34) |
| 139 | Republic of Namibia | 10.93 (0 to 47.82) | 225.24 (0.11 to 946.54) | -1.14 (-1.38 to -0.89) | -1.38 (-1.65 to -1.1) |
| 140 | Republic of Nauru | 69.32 (7.3 to 167.42) | 1450.34 (142.3 to 3634.5) | -0.69 (-0.99 to -0.39) | -0.57 (-0.93 to -0.21) |
| 141 | Republic of Nicaragua | 14.99 (0.91 to 38.81) | 314.31 (20.84 to 812.64) | -0.36 (-0.57 to -0.16) | -0.35 (-0.54 to -0.17) |
| 142 | Republic of Niue | 42.65 (4.52 to 98.49) | 836.85 (91.13 to 1951.11) | -0.87 (-0.96 to -0.78) | -0.8 (-0.92 to -0.68) |
| 143 | Republic of Palau | 37.14 (3.73 to 88) | 737.98 (71.63 to 1813.33) | -0.72 (-0.77 to -0.66) | -0.7 (-0.76 to -0.63) |
| 144 | Republic of Panama | 12.36 (0.67 to 31.61) | 255.03 (15.62 to 658.47) | -1.17 (-1.33 to -1.01) | -1.1 (-1.26 to -0.95) |
| 145 | Republic of Paraguay | 16.55 (0.56 to 47) | 327.92 (11.79 to 917.13) | -0.74 (-0.9 to -0.58) | -0.85 (-0.96 to -0.73) |
| 146 | Republic of Peru | 9.28 (0.21 to 27.94) | 189.2 (4.64 to 583.28) | -2.26 (-2.64 to -1.88) | -2.16 (-2.53 to -1.79) |
| 147 | Republic of Poland | 26.05 (4.7 to 54.51) | 461.14 (79.2 to 981.41) | -2.66 (-2.88 to -2.44) | -2.52 (-2.76 to -2.27) |
| 148 | Republic of Rwanda | 27.08 (0.9 to 72.87) | 496.11 (16.64 to 1325.4) | -3.9 (-4.31 to -3.49) | -4.36 (-4.79 to -3.92) |
| 149 | Republic of San Marino | 3.12 (0.01 to 12.3) | 64.84 (0.53 to 247.51) | -2.41 (-2.78 to -2.04) | -2.22 (-2.51 to -1.92) |
| 150 | Republic of Senegal | 14.91 (0.01 to 54.63) | 302.82 (0.23 to 1110.27) | 0.11 (0.05 to 0.16) | -0.05 (-0.12 to 0.02) |
| 151 | Republic of Serbia | 64.13 (18.97 to 118.08) | 1076.64 (337.59 to 1961.91) | -2.77 (-3 to -2.53) | -2.81 (-3.02 to -2.61) |
| 152 | Republic of Seychelles | 35.12 (4.49 to 76.56) | 670.47 (96.48 to 1463) | -2.04 (-2.18 to -1.9) | -2.39 (-2.55 to -2.23) |
| 153 | Republic of Sierra Leone | 14.51 (0.02 to 53.07) | 306.31 (0.36 to 1136.81) | 0.44 (0.2 to 0.67) | 0.4 (0.17 to 0.63) |
| 154 | Republic of Singapore | 10.07 (1.55 to 21.41) | 220.07 (39.3 to 451.51) | -4.35 (-4.45 to -4.25) | -4.36 (-4.46 to -4.26) |
| 155 | Republic of Slovenia | 20.38 (5.8 to 36.84) | 343.1 (103.28 to 632.26) | -3.16 (-3.31 to -3.01) | -3.77 (-3.91 to -3.62) |
| 156 | Republic of South Africa | 7.34 (0 to 31.9) | 153.5 (0.11 to 646.41) | -0.27 (-0.63 to 0.1) | -0.86 (-1.19 to -0.53) |
| 157 | Republic of South Sudan | 27.06 (0.85 to 69.99) | 533.12 (17.37 to 1428.42) | -1.94 (-2.21 to -1.66) | -2.05 (-2.34 to -1.75) |
| 158 | Republic of Sudan | 8.47 (0 to 43.99) | 191.07 (0.04 to 933.84) | -1.3 (-1.35 to -1.24) | -1.43 (-1.48 to -1.37) |
| 159 | Republic of Suriname | 12.94 (0.06 to 42.62) | 262.06 (1.1 to 897.66) | -1.41 (-1.61 to -1.21) | -1.28 (-1.49 to -1.07) |
| 160 | Republic of Tajikistan | 34.75 (2.39 to 90.85) | 583.58 (33.5 to 1626.76) | -2.21 (-2.54 to -1.89) | -2.57 (-2.85 to -2.3) |
| 161 | Republic of Trinidad and Tobago | 11.56 (0.05 to 39.12) | 242.84 (1.17 to 814.78) | -2.45 (-2.67 to -2.22) | -2.25 (-2.48 to -2.02) |
| 162 | Republic of Tunisia | 5.52 (0 to 30.1) | 112.7 (0.02 to 564.31) | -1.21 (-1.3 to -1.12) | -1.25 (-1.33 to -1.17) |
| 163 | Republic of Turkey | 3.5 (0 to 21.81) | 70.27 (0 to 416.24) | -1.88 (-2.24 to -1.52) | -2.36 (-2.66 to -2.06) |
| 164 | Republic of Uganda | 21.26 (0.7 to 57.07) | 404.77 (12.88 to 1119.07) | -2.19 (-2.43 to -1.95) | -2.47 (-2.74 to -2.2) |
| 165 | Republic of Uzbekistan | 35.41 (1.96 to 91.06) | 626.36 (30.54 to 1682.52) | -1.17 (-1.41 to -0.93) | -1.53 (-1.75 to -1.31) |
| 166 | Republic of Vanuatu | 52.11 (5.43 to 122.3) | 1070.38 (110.45 to 2635.11) | -0.78 (-0.85 to -0.7) | -0.65 (-0.75 to -0.56) |
| 167 | Republic of Yemen | 10.03 (0 to 55.59) | 219.55 (0.02 to 1163.35) | -1.12 (-1.23 to -1.01) | -1.36 (-1.47 to -1.24) |
| 168 | Republic of Zambia | 38.65 (1.4 to 99.05) | 705.99 (28.17 to 1874.05) | -1.43 (-1.59 to -1.27) | -1.7 (-1.87 to -1.53) |
| 169 | Republic of Zimbabwe | 16.71 (0.03 to 61.24) | 368.35 (0.8 to 1345.06) | 1.5 (1.05 to 1.95) | 1.69 (1.18 to 2.2) |
| 170 | Republic of the Congo | 18.65 (0.03 to 69.44) | 329.52 (0.38 to 1309.18) | -0.46 (-0.65 to -0.26) | -0.68 (-0.87 to -0.49) |
| 171 | Republic of the Gambia | 16.77 (0.02 to 62.92) | 345.59 (0.4 to 1300.48) | 0.73 (0.6 to 0.86) | 0.58 (0.43 to 0.73) |
| 172 | Republic of the Marshall Islands | 54.59 (6.15 to 129.26) | 1102.5 (124.82 to 2733.8) | -0.67 (-0.76 to -0.57) | -0.54 (-0.66 to -0.41) |
| 173 | Republic of the Niger | 11.99 (0.01 to 45.13) | 247.3 (0.21 to 940.46) | 0.1 (0.04 to 0.16) | -0.04 (-0.11 to 0.03) |
| 174 | Republic of the Philippines | 44.17 (8.13 to 92.1) | 981.24 (193.63 to 2054.97) | -0.8 (-0.86 to -0.75) | -0.77 (-0.85 to -0.7) |
| 175 | Republic of the Union of Myanmar | 46.73 (7.15 to 101.31) | 999.31 (164.76 to 2162.34) | -2.51 (-2.63 to -2.38) | -2.86 (-3 to -2.72) |
| 176 | Romania | 57.95 (16.83 to 106.93) | 1050.77 (317.8 to 1941.84) | -2.42 (-2.57 to -2.27) | -2.59 (-2.77 to -2.41) |
| 177 | Russian Federation | 22.22 (1.24 to 64.18) | 506.05 (38.43 to 1358.8) | -1.81 (-2.32 to -1.29) | -1.86 (-2.43 to -1.29) |
| 178 | Saint Kitts and Nevis | 16.91 (0.09 to 54.02) | 302.23 (1.25 to 997.13) | -1.71 (-1.94 to -1.49) | -1.78 (-2.03 to -1.52) |
| 179 | Saint Lucia | 12.95 (0.08 to 41.71) | 227.37 (1.3 to 756.91) | -3.06 (-3.49 to -2.63) | -2.62 (-2.98 to -2.25) |
| 180 | Saint Vincent and the Grenadines | 20.18 (0.17 to 60.98) | 345.66 (2.14 to 1094.97) | -1.02 (-1.32 to -0.72) | -0.97 (-1.26 to -0.68) |
| 181 | Slovak Republic | 44.92 (13.79 to 85.28) | 806.09 (253.12 to 1523.14) | -2.3 (-2.37 to -2.23) | -2.75 (-2.83 to -2.67) |
| 182 | Socialist Republic of Viet Nam | 40.75 (6.01 to 86.43) | 840.85 (136.6 to 1776.58) | -0.99 (-1.06 to -0.92) | -1.17 (-1.25 to -1.09) |
| 183 | Solomon Islands | 53.52 (6.62 to 125.93) | 1071.14 (121.94 to 2618.75) | -0.77 (-0.85 to -0.69) | -0.65 (-0.73 to -0.56) |
| 184 | State of Eritrea | 36.45 (1.5 to 92.35) | 687.3 (28.54 to 1809.31) | -1.85 (-1.95 to -1.74) | -2.15 (-2.27 to -2.03) |
| 185 | State of Israel | 3.51 (0.02 to 12.26) | 71.26 (0.56 to 239.09) | -4.02 (-4.21 to -3.83) | -3.78 (-3.99 to -3.58) |
| 186 | State of Kuwait | 7.15 (0.02 to 25.98) | 152.16 (0.88 to 522.8) | -1.87 (-2.33 to -1.41) | -2.03 (-2.47 to -1.59) |
| 187 | State of Libya | 5.93 (0 to 31.61) | 134.23 (0.03 to 670.39) | 0.62 (0.44 to 0.81) | 0.54 (0.36 to 0.72) |
| 188 | State of Qatar | 3.63 (0 to 19.84) | 73.72 (0.01 to 373.61) | -4.22 (-4.91 to -3.52) | -4.07 (-4.68 to -3.46) |
| 189 | Sultanate of Oman | 6.43 (0 to 34.18) | 133.66 (0.02 to 672.4) | -1.16 (-1.33 to -0.98) | -1.48 (-1.62 to -1.34) |
| 190 | Swiss Confederation | 3.94 (0.02 to 13.89) | 67.98 (0.61 to 229.94) | -2.65 (-2.72 to -2.59) | -2.93 (-3.03 to -2.84) |
| 191 | Syrian Arab Republic | 8.67 (0 to 47.33) | 178.3 (0.02 to 912.64) | -0.99 (-1.16 to -0.82) | -1.3 (-1.49 to -1.11) |
| 192 | Taiwan (Province of China) | 11.57 (1.27 to 26.38) | 210.8 (19.77 to 508.28) | -3.3 (-3.64 to -2.95) | -3.34 (-3.65 to -3.03) |
| 193 | Togolese Republic | 15.63 (0.02 to 59.06) | 328.75 (0.35 to 1209.1) | 0.35 (0.16 to 0.54) | 0.27 (0.07 to 0.47) |
| 194 | Tokelau | 37.19 (3.97 to 84.27) | 723.93 (72.98 to 1698.25) | -1.55 (-1.62 to -1.48) | -1.45 (-1.55 to -1.36) |
| 195 | Turkmenistan | 39.08 (2.18 to 107.88) | 746.7 (37.02 to 2107.79) | -2.16 (-2.5 to -1.82) | -2.13 (-2.45 to -1.81) |
| 196 | Tuvalu | 48.86 (5.67 to 112.87) | 974.68 (106.13 to 2352.11) | -1.17 (-1.22 to -1.12) | -1.09 (-1.14 to -1.03) |
| 197 | Ukraine | 13.97 (0.02 to 57.34) | 288.66 (0.68 to 1200.41) | -1.29 (-1.7 to -0.87) | -1.22 (-1.63 to -0.81) |
| 198 | Union of the Comoros | 25.73 (0.91 to 66.51) | 487.55 (18.33 to 1264.82) | -2.2 (-2.41 to -1.99) | -2.43 (-2.65 to -2.21) |
| 199 | United Arab Emirates | 5.75 (0 to 32.54) | 111.98 (0.01 to 591.08) | 0.01 (-0.51 to 0.54) | -0.89 (-1.32 to -0.45) |
| 200 | United Kingdom of Great Britain and Northern Ireland | 3.68 (0.03 to 13.11) | 68.81 (0.76 to 242.21) | -3.41 (-3.58 to -3.24) | -3.32 (-3.52 to -3.12) |
| 201 | United Mexican States | 11.49 (0.22 to 34.34) | 225.91 (5.15 to 680.06) | -0.71 (-0.83 to -0.58) | -0.46 (-0.56 to -0.37) |
| 202 | United Republic of Tanzania | 35.16 (3.28 to 78.77) | 656.73 (59.56 to 1510.84) | -0.69 (-0.76 to -0.63) | -0.92 (-0.99 to -0.85) |
| 203 | United States Virgin Islands | 9.93 (0.06 to 31.47) | 179.86 (1.02 to 582.63) | -2.35 (-2.52 to -2.18) | -2.2 (-2.36 to -2.04) |
| 204 | United States of America | 7.93 (0.19 to 24.69) | 185.47 (6.39 to 523.22) | -0.12 (-0.23 to 0) | 0.48 (0.34 to 0.62) |

**Supplementary Table S4.** The BAPC model's prediction of ASDR and ASYR attributable to high-sodium diets by different genders from 2022 to 2040.

|  | **ASDR** | | | **ASYR** | | |
| --- | --- | --- | --- | --- | --- | --- |
| **year** | **Male** | **Female** | **Both** | **Male** | **Female** | **Both** |
| 2022 | 29.83 | 14.77 | 21.52 | 659.16 | 304.41 | 471.83 |
| 2023 | 29.43 | 14.59 | 21.24 | 651.38 | 300.91 | 466.55 |
| 2024 | 29.04 | 14.4 | 20.95 | 643.61 | 297.35 | 461.23 |
| 2025 | 28.64 | 14.22 | 20.67 | 635.8 | 293.73 | 455.86 |
| 2026 | 28.26 | 14.03 | 20.39 | 628.16 | 290.19 | 450.65 |
| 2027 | 27.88 | 13.84 | 20.12 | 620.84 | 286.76 | 445.67 |
| 2028 | 27.51 | 13.66 | 19.85 | 613.71 | 283.39 | 440.78 |
| 2029 | 27.15 | 13.48 | 19.59 | 606.6 | 279.98 | 435.84 |
| 2030 | 26.79 | 13.3 | 19.33 | 599.44 | 276.51 | 430.86 |
| 2031 | 26.44 | 13.12 | 19.07 | 592.46 | 273.13 | 426.03 |
| 2032 | 26.1 | 12.94 | 18.82 | 585.78 | 269.85 | 421.43 |
| 2033 | 25.77 | 12.77 | 18.58 | 579.24 | 266.6 | 416.86 |
| 2034 | 25.44 | 12.59 | 18.34 | 572.64 | 263.29 | 412.2 |
| 2035 | 25.12 | 12.42 | 18.1 | 565.96 | 259.93 | 407.47 |
| 2036 | 24.81 | 12.24 | 17.86 | 559.41 | 256.65 | 402.88 |
| 2037 | 24.51 | 12.08 | 17.64 | 553.17 | 253.48 | 398.51 |
| 2038 | 24.21 | 11.91 | 17.42 | 547.05 | 250.34 | 394.18 |
| 2039 | 23.92 | 11.74 | 17.2 | 540.85 | 247.13 | 389.74 |
| 2040 | 23.62 | 11.58 | 16.97 | 534.53 | 243.88 | 385.21 |
